# Supplementary material for: Capturing single-cell heterogeneity via data fusion improves image-based profiling
Source: Nat Commun. 2019 May 7;10:2082. doi: 10.1038/s41467-019-10154-8 (PMC6504923; doi:10.1038/s41467-019-10154-8)
Supplement: Supplementary file 1 — Supplementary Information [file 41467_2019_10154_MOESM1_ESM.pdf]

Capturing single-cell heterogeneity via data fusion improves image-based profiling

Rohban et al.

Supplementary Figures and Tables

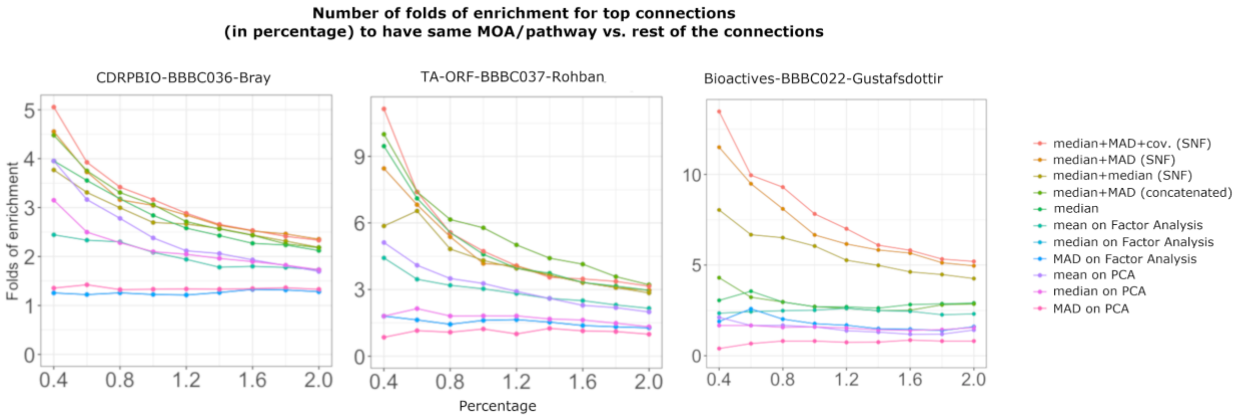

Supplementary Figure 1: The profiles defined based on factor analysis or principal component analysis give lower enrichment score than the proposed method of fusing similarity scores arising from median, MAD, and random projection of covariances. MOAs that have fewer than 5 compound pairs are filtered out in the analysis to ensure that the improvement in each method is not driven by noisy improvements in MOAs with few members.

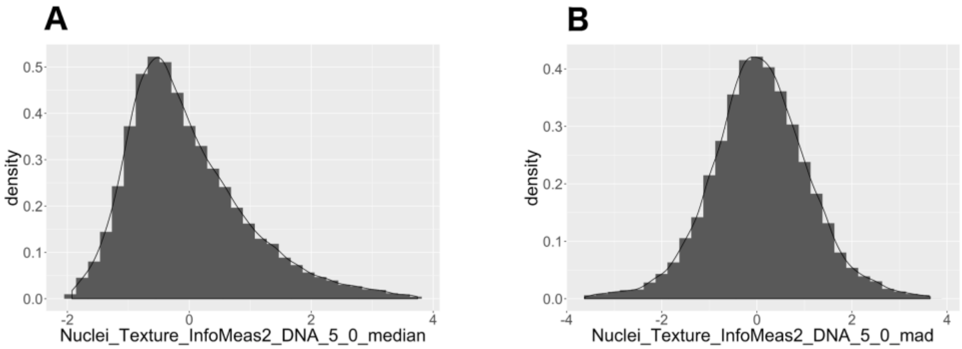

Supplementary Figure 2: Features may show different distributions in median and MAD profiles. (A) shows that a texture feature in the DNA channel in median profiles has a skewed distribution in CDRP-BBBC036-Bray. On the other hand, MAD profiles gives a nearly symmetric distribution for the same feature (B). Values lower than first and higher than 99th percentiles are removed before plotting the distributions.

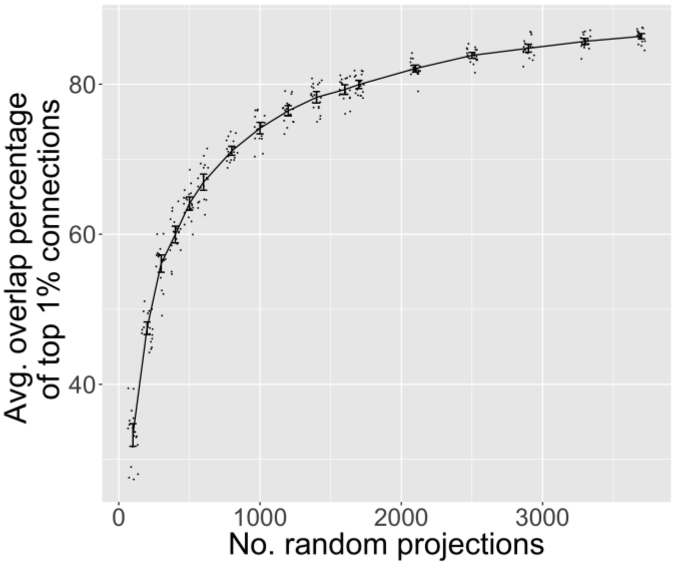

Supplementary Figure 3: Top-correlated treatment pairs become increasingly consistent as number of random projections in increased. Average percentage of overlap size between top 1% correlated treatment pairs between two random projections increases sharply and saturates around 3000 random projections. The data is taken from a single plate in the CDRP-BBBC036-Bray dataset and the solid line represents the average of 20 independent random simulations, where each dot shows the outcome of a simulation. Error bars represent standard deviation of the simulation outcomes.

Supplementary Table 1: The proposed median+MAD+covariance (data-fused) profiles significantly improve recall vs. state-of-the-art median+MAD (concatenated). More specifically, the percentage of same MOA/pathway connections that are captured in the top 0.5% most-similar treatment pairs is significantly higher in median+MAD+covariance (data-fused) profiles compared to median+MAD (concatenated) profiles in CDRPBIO-BBBC036-Bray and Bioactives-BBBC022-Gustafsdottir (Fisher's test). The number of genes or compounds in the experiment is indicated by n in the table.

Dataset	95% confidence interval of the odds ratio	Relative increase in validated hits (1 - odds ratio)	P-value (One sided Fisher's test)
CDRPBIO-BBBC036-Bray (n compounds = 1552)	(1.013, Inf)	19%	0.038
Bioactives-BBBC022-Gustafsdottir (n compounds = 1048)	(1.861, Inf)	132%	1.227e-11
TA-ORF-BBBC037-Rohban (n genes = 205)	(0.736, Inf)	12%	0.36

Supplementary Table 2: The proposed median+MAD+covariance (data-fused) profiles significantly improve precision vs. state-of-the-art median+MAD (concatenated). More specifically, the top 0.5% most-similar treatment pairs contain more same MOA/pathway pairs in median+MAD+covariance (data-fused) profiles compared to median+MAD (concatenated) profiles in CDRPBIO-BBBC036-Bray and Bioactives-BBBC022-Gustafsdottir (Fisher's test). The number of genes or compounds in the experiment is indicated by n in the table.

Dataset	95% confidence interval of the odds ratio	Relative increase in validated hits (1 - odds ratio)	P-value (One sided Fisher's test)
CDRPBIO-BBBC036-Bray (n compounds = 1552)	(1.015, Inf)	20%	0.036
Bioactives-BBBC022-Gustafsdottir (n compounds = 1048)	(1.921, Inf)	140%	4.458e-12
TA-ORF-BBBC037-Rohban (n genes = 205)	(0.705, Inf)	18%	0.33

Supplementary Table 3: Sorted list of MOAs based on improvement of median+MAD+cov. (SNF) compared to state-of-the-art median+MAD (concatenated) in CDRPBIO-BBBC036-Bray. TGF-beta receptor inhibitors, ATP channel blockers, Tubulin inhibitors, and Glycogen synthase kinase inhibitors are among the MOAs showing improvements.

MOA Name	Percentage of same-MOA pairs captured in top 0.5% most-similar connections		Total number of same-MOA pairs
	median+MAD+ cov. (SNF)	median+MAD (concatenated)	
rho associated kinase inhibitor	67	0	3
proteasome inhibitor	100	50	2
pka inhibitor	50	0	2
tgf beta receptor inhibitor|p38 mapk inhibitor	50	0	2
tgf beta receptor inhibitor	29	0	7
microtubule inhibitor|tubulin inhibitor	100	75	4
atp channel blocker	20	0	5
adrenergic receptor antagonist|serotonin receptor antagonist	17	0	12
pi3k inhibitor	29	14	7
cdk inhibitor|cftr channel activator|glycogen synthase kinase inhibitor|jnk inhibitor	14	0	7
glycogen synthase kinase inhibitor	13	0	15
retinoid receptor agonist	20	8	25
p38 mapk inhibitor	18	6	51

cannabinoid receptor antagonist	8	0	13
bacterial dna gyrase inhibitor	5	0	19
cdk inhibitor	8	3	78
opioid receptor antagonist	4	0	24
dopamine receptor antagonist|serotonin receptor antagonist	3	0	36
atpase inhibitor	40	38	77
cytochrome p450 inhibitor	1	0	74
serotonin receptor agonist	1	0	453
dopamine receptor antagonist	1	1	1198
adrenergic receptor agonist	1	1	521
tubulin polymerization inhibitor	96	96	24
hmgcr inhibitor	36	36	14
microtubule inhibitor	100	100	3
topoisomerase inhibitor	12	12	25
acetylcholinesterase inhibitor|microtubule inhibitor|tubulin inhibitor	100	100	2

estrogen receptor agonist	2	2	90
protein synthesis inhibitor	4	4	52
antiamyloidogenic agent	25	25	4
bacterial permeability inducer	100	100	1
cdc inhibitor	50	50	2
dehydrogenase inhibitor|inositol monophosphatase inhibitor	100	100	1
dna dependent protein kinase inhibitor|mtor inhibitor|pi3k inhibitor	100	100	1
microtubule inhibitor|tubulin polymerization inhibitor	100	100	1
protein phosphatase inhibitor	100	100	1
src inhibitor	5	5	20
vitamin d receptor agonist	100	100	1
serotonin receptor antagonist	1	1	1019
egfr inhibitor	2	2	189
glutamate receptor antagonist	1	1	346
dopamine receptor agonist	0	2	321

tyrosine kinase inhibitor	2	3	61
acetylcholine receptor agonist	0	2	57
sodium channel blocker	0	2	251
glucocorticoid receptor agonist	16	18	237
dopamine uptake inhibitor	5	11	19
pkc inhibitor	0	7	14
dopamine receptor agonist|serotonin receptor antagonist	0	8	13
serotonin receptor antagonist|collagen stimulant	0	11	9
egfr inhibitor|src inhibitor	0	12	8
hdac inhibitor	14	29	7
bacterial 50s ribosomal subunit inhibitor	0	20	5
egfr inhibitor|erbb2 inhibitor|jak2 inhibitor	0	25	4
egfr inhibitor|epidermal growth factor receptor (egfr) inhibitor|tyrosine kinase inhibitor	0	100	1

Supplementary Table 4: Sorted list of MOAs based on improvement of median+MAD+cov. (SNF) compared to state-of-the-art median+MAD (concatenated) in Bioactives-BBBC022-Gustafsdottir. Tubulin inhibitors and Glucocorticoid receptor agonists are among the MOAs showing improvements.

MOA Name	Percentage of same-MOA pairs captured in top 0.5% most-similar connections		Total number of same-MOA pairs
	median+MAD+ cov. (SNF)	median+MAD (concatenated)	
trpv agonist|cannabinoid receptor agonist	100	0	1
tubulin polymerization inhibitor	75	38	16
anthelmintic agent	33	0	3
serotonin reuptake inhibitor	25	0	4
glucocorticoid receptor agonist	18	2	485
protein synthesis inhibitor	23	8	13
atpase inhibitor	23	10	93
nfkb pathway inhibitor	12	0	8
topoisomerase inhibitor	7	0	29
dopamine receptor agonist	2	0	243
estrogen receptor agonist	2	0	53
phosphodiesterase inhibitor	1	0	93
serotonin receptor antagonist	1	0	500

dopamine receptor antagonist	3	2	1027
adrenergic receptor antagonist	1	1	438
microtubule inhibitor	67	67	3
calcineurin inhibitor	100	100	1
cytochrome p450 inhibitor	5	5	20
dopamine receptor agonist|serotonin receptor antagonist	10	10	10
tubulin inhibitor	50	50	2
serotonin receptor agonist	0	1	149
acetylcholine receptor antagonist	1	2	277
calcium channel blocker	0	1	219
dopamine receptor antagonist|serotonin receptor antagonist	0	2	65
monoamine oxidase inhibitor	0	2	54
bacterial cell wall synthesis inhibitor	1	3	176
anti-hcve2	0	100	1

Supplementary Table 5: Sorted list of pathways based on improvement of median+MAD+cov. (SNF) compared to state-of-the-art median+MAD (concatenated) in TA-ORF-BBBC037-Rohban. PKC, TGF-beta, Hippo, and NOTCH are among the pathways showing improvements.

Pathway Name	Percentage of same-pathway pairs captured in top 0.5% most-similar connections		Total number of same-pathway pairs
	median+MAD+cov. (SNF)	median+MAD (concatenated)	
pkc	33	0	3
tgfbeta	17	0	6
hippo	24	14	21
notch	4	0	28
hypoxia	2	0	45
er stress/upr	2	0	66
pi3k/akt	1	0	78
tor	1	1	171
hedgehog	33	33	3
insulin receptor signaling	17	17	6
pka	3	3	36

transcription factors	100	100	1
wnt	1	1	78
mapk	5	6	378
cytoskeletal re-org	0	10	10
rtk	0	33	3
